# Invasion of Diffuse Large B-Cell Lymphoma to Right Axillary Arterial Graft

**DOI:** 10.3400/avd.cr.24-00132

**Published:** 2025-03-25

**Authors:** Koki Yokawa, Yukihiro Imai, Taku Nakagawa, Makoto Kusakizako, Yosuke Tanaka, Tomonori Higuma, Kazunori Yoshida, Yoshihiro Oshima, Hidefumi Obo, Hidetaka Wakiyama

**Affiliations:** 1 Department of Cardiovascular Surgery, Kakogawa Central City Hospital, Kakogawa, Hyogo, Japan; 2 Department of Diagnostic Pathology, Kakogawa Central City Hospital, Kakogawa, Hyogo, Japan

**Keywords:** diffuse large B-cell lymphoma, thoracic endovascular aortic repair, bypass graft

## Abstract

A 76-year-old male patient, who had undergone right axillary artery bypass and arch replacement surgery for retrograde type A aortic dissection after thoracic endovascular aortic repair 2 years ago, was referred to our department with complaints of swelling and pain in the right subclavian region. A computed tomography scan suspected an abscess around the bypass graft; however, the culture was negative. Pathological examination indicated a diffuse large B-cell lymphoma (DLBCL) diagnosis. Chemotherapy was not indicated due to the patient’s condition, and he passed away after 3 months. DLBCL originating around a graft is extremely rare but crucial for differential diagnosis.

## Introduction

Diffuse large B-cell lymphoma (DLBCL) is the most representative type of aggressive lymphoma and is known to metastasize throughout the body. However, metastasis to the arterial system is extremely rare.

## Case Report

A 76-year-old male patient was referred to our department with a chief complaint of swelling and pain in the right anterior chest that persisted for 1 week. He had undergone zone 2 thoracic endovascular aortic repair (TEVAR) and left common carotid artery to left axillary artery bypass surgery for a thoracic aortic aneurysm 6 years ago. He had an aortic valve replacement and tricuspid annuloplasty for aortic regurgitation and tricuspid regurgitation 5 years ago. He underwent right axillary artery to ascending aorta bypass surgery and partial arch replacement for retrograde type A aortic dissection after TEVAR in our department 2 years ago. The graft used for the bypass to the right axillary artery was the 8 mm side branch of J Graft (Japan Lifeline Co., Ltd., Tokyo, Japan) used for the partial arch replacement. The patient had chronic atrial fibrillation, in addition to the aortic and cardiac surgeries, and was on anticoagulant therapy. Additionally, he received radiation therapy for laryngeal cancer 12 years ago. An adenoma was found in the left thyroid lobe, and he had hypothyroidism, which was under follow-up.

**Figure 1:**
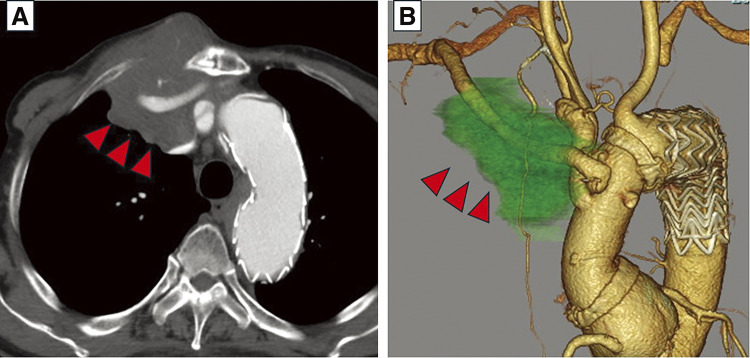
Fig. 1 Preoperative CT revealing a mass surrounding the graft to the right axillary artery. (**A**) Axial CT imaging showing tumor progression (indicated by red arrowheads). (**B**) 3D CT image highlighting the tumor with red arrowheads and light green coloring. CT: computed tomography

**Figure 2:**
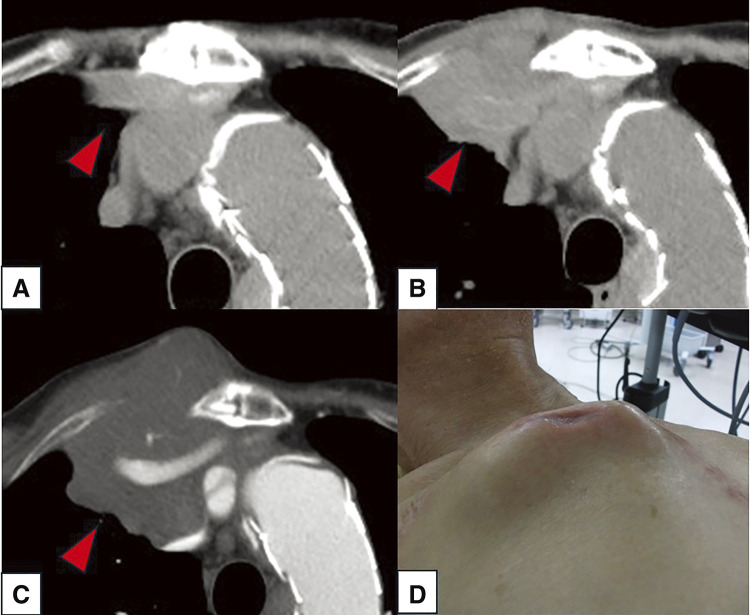
Fig. 2 Changes in the mass on preoperative computed tomography scan. The red arrowhead indicates the enlargement of the anterior mediastinal mass during this course. (**A**) 13 months before admission, (**B**) 1 month before admission, (**C**) current admission, and (**D**) preoperative external findings.

The laboratory test revealed only an elevated inflammatory response (C-reactive protein: 2.45 mg/dL) and mild renal dysfunction (creatinine:1.13 mg/dL; estimated glomerular filtration rate: 49%). The white blood cell count was 6333/mL, with monocytes accounting for 13.3%. Lactate dehydrogenase and alkaline phosphatase levels were elevated, measuring 308 and 409 U/L, respectively. Trans-thoracic echocardiography detected left ventricular enlargement (LVDd/Ds: 56.4 mm/39.3 mm), moderate mitral insufficiency, and a decreased ejection fraction (48%). No prosthetic valve dysfunction was observed. Computed tomography (CT) examination revealed a rapidly enlarging mass around the prosthetic graft in the right anterior chest (**Figs. 1** and **2**).

**Figure 3:**
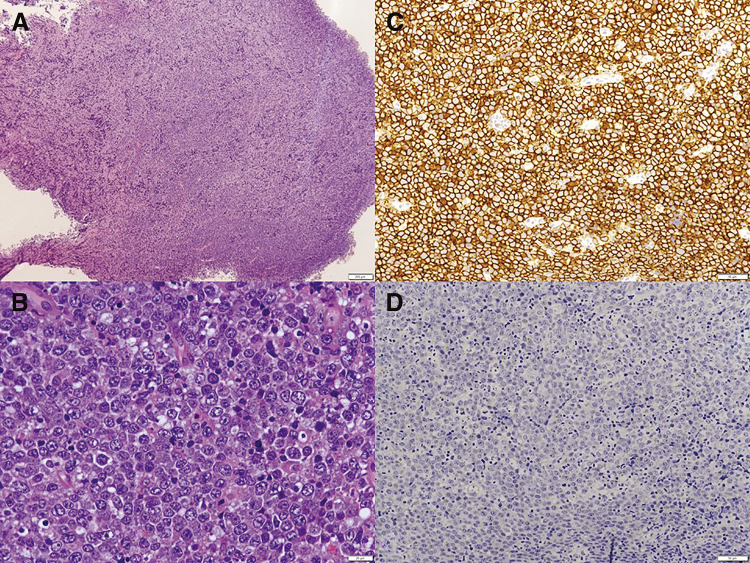
Fig. 3 Pathological findings. (**A**) HE stain (×4), (**B**) HE stain (×50), (**C**) CD20 (×20), and (**D**) EB virus-encoded small RNA (×20). Immunohistochemistry showed positive staining for CD20, and EB virus encoded small RNA in situ hybridization was negative. HE: hematoxylin and eosin; EB: Epstein–Barr; RNA: ribonucleic acid

CT examination could not rule out a prosthetic graft infection; thus, we decided to proceed with urgent open drainage surgery of the anterior chest. Intraoperative results revealed no obvious abscess formation, but a grayish-white, friable, gelatinous mass. Completely resecting this tumorous lesion was challenging; hence, the wound was managed in an open state until obtaining a definitive diagnosis. Bacterial infection was ruled out, but findings were indicative of malignant lymphoma. The bacterial culture of the excised specimen was negative, and pathological examination confirmed a DLBCL diagnosis. Histopathological examination of resected tissue indicated diffuse infiltration of large lymphocytes with granular chromatin and scattered mitoses. Small lymphocytes and nuclear dust were scattered between large lymphocytes. Immunohistochemistry revealed positive staining for CD20, bcl2, and MUM1, whereas CD3, CD10, CD30, and bcl6 were negative. These results revealed that the tumor was DLBCL non-germinal center B-cell type. Epstein–Barr (EB) virus-encoded small RNA in situ hybridization was negative (**Fig. 3**). The tumorous lesion in the anterior chest revealed a tendency to enlarge during the postoperative course. No results indicated metastasis to other organs. The clinical stage was deemed to involve local infiltration when consulted with a hematologist, equivalent to stage IV. The patient’s declining performance status during hospitalization, decreased cardiac function, chronic kidney disease, and other organ impairments increased the risk of complications from chemotherapy while indicating curative chemotherapy. Full-dose treatment was difficult, thereby decreasing the likelihood of disease cure. The best supportive care approach was adopted after discussions with the patient and family. The patient passed away 3 months post-drainage surgery.

## Discussion

Primary malignant lymphoma originating from the chest wall accounts for approximately 0.3%–1.0% of extranodal lymphoma. This lymphoma is mostly associated with underlying diseases, such as tuberculous pleurisy or pyothorax after artificial pneumothorax, and is caused by pleural chronic inflammation.^1)^ However, the present case demonstrated none of these predominant features. In this case, the lymphoma had progressed along the artificial graft, and chronic inflammation caused by the artificial graft cannot be completely ruled out as contributing to malignant lymphoma development. Breast implant-associated anaplastic large cell lymphoma is reported as a lymphoma related to artificial materials. The biofilm surrounding the textured implants may stimulate lymphocyte production, which triggers an inflammation cycle that ultimately causes breast implant-associated anaplastic large cell lymphoma.^2)^ However, the pathological results in this case do not match this lymphoma subtype. The cause remains unclear because no reports describe malignant lymphoma occurring around artificial grafts, such as in this case.

If a biopsy via puncture had been considered as a preoperative diagnostic method, the diagnosis could have been made more quickly. Unfortunately, we were unable to perform a postmortem autopsy, and therefore, we could not confirm any pathological findings of malignant lymphoma infiltration into the graft.

Previous reports have described favorable outcomes with aggressive treatment for malignant lymphomas originating from the aorta or heart.^3,4)^ However, chemotherapy was invariably administered in these cases, whereas the patient in our case was unable to undergo chemotherapy and subsequently passed away. This case experienced multiple comorbidities, but the prognosis may have been prolonged if chemotherapy had been administered.

## Conclusion

We present a novel case of DLBCL with the appearance of an infected graft after thoracic aortic surgery. DLBCL originating around artificial graft is extremely rare but crucial for differential diagnosis. Aggressive chemotherapy is considered necessary when diagnosing DLBCL if a favorable prognosis is expected.

## Declarations

### Consent for publication

We have obtained the patient’s consent for publication.

### Ethics approval

This study was approved by the Institutional Review Board with approval number 2024-22.

### Funding

No funding was received for this study.

### Disclosure statement

The authors have nothing to disclose.

### Author contributions

Manuscript preparation: KY

Critical review and revision: all authors

Final approval of the article: all authors

Accountability for all aspects of the work: all authors.
